# The role of the gut microbiota in neurodegenerative diseases targeting metabolism

**DOI:** 10.3389/fnins.2024.1432659

**Published:** 2024-09-26

**Authors:** Yufeng Fu, Zhongya Gu, Huan Cao, Chengchao Zuo, Yaqi Huang, Yu Song, Yongsheng Jiang, Furong Wang

**Affiliations:** ^1^Department of Neurology, Tongji Medical College, Tongji Hospital, Huazhong University of Science and Technology, Wuhan, Hubei, China; ^2^Department of Rehabilitation, Tongji Medical College, Tongji Hospital, Huazhong University of Science and Technology, Wuhan, Hubei, China; ^3^Cancer Center of Tongji Hospital, Tongji Medical College, Huazhong University of Science and Technology, Wuhan, Hubei, China; ^4^Key Laboratory of Vascular Aging (HUST), Ministry of Education, Tongji Medical College, Tongji Hospital, Huazhong University of Science and Technology, Wuhan, Hubei, China

**Keywords:** neurodegenerative diseases, gut microbiota, metabolism, therapy, fecal microbiota transplantation

## Abstract

In recent years, the incidence of neurodegenerative diseases (NDs) has gradually increased over the past decades due to the rapid aging of the global population. Traditional research has had difficulty explaining the relationship between its etiology and unhealthy lifestyle and diets. Emerging evidence had proved that the pathogenesis of neurodegenerative diseases may be related to changes of the gut microbiota’s composition. Metabolism of gut microbiota has insidious and far-reaching effects on neurodegenerative diseases and provides new directions for disease intervention. Here, we delineated the basic relationship between gut microbiota and neurodegenerative diseases, highlighting the metabolism of gut microbiota in neurodegenerative diseases and also focusing on treatments for NDs based on gut microbiota. Our review may provide novel insights for neurodegeneration and approach a broadly applicable basis for the clinical therapies for neurodegenerative diseases.

## 1 Introduction

Neurodegenerative diseases, which constitute a complex and heterogeneous group of conditions, are characterized at their core by a progressive and irreversible degeneration of the structure and function of the nervous system. At a time of accelerated global population ageing, the incidence of these diseases has steadily increased over the past decades (Brayne and Miller, 2017; Milo and Kahana, 2010). Alzheimer’s disease (AD), Parkinson’s disease (PD), Huntington’s disease (HD), and multiple sclerosis (MS) are the most common neurodegenerative diseases. The primary pathological hallmarks of NDs are chronic and progressive loss of neurons, which is caused by the deposition of neurotoxic etiological substances in the central nervous system (Tang and Le, 2016). There are still many gaps to fill in curing neurodegenerative diseases despite the numerous advances made to understand the complex mechanisms in the past decades.

Bacteria, archaea, viruses, and various eukaryotes, a variety of microorganisms, form the gut microbiota, which exists in different niches of the intestine (Arumugam et al., 2011). It is established that the overall health of humans is closely affected by the gut microbiota (Gates et al., 2022). The host physiology, including aspects of nutrient metabolism, neuroinflammation, neuroimmunology and neurodevelopment, can be influenced profoundly by gut microbiota (Sommer and Bäckhed, 2013; Geva-Zatorsky et al., 2017). As is widely recognized, the gut microbiota plays an integral role in promoting our overall health. This includes safeguarding us against pathogens, aiding in nutrient absorption and synthesis, facilitating metabolic processes, and modulating immune responses, among other essential functions (Nishida et al., 2018). Interestingly, Kerstin Berer found that gut microbiota is associated with neurodegenerative diseases, which made research on the correlation between neurodegenerative diseases and gut microbiota become a hot topic (Berer et al., 2017). What’s more, aging is an essential and important factor in the progression of neurodegenerative diseases, which is a natural process that is influenced by various biological and genetic mechanisms. The effects of microbial composition and associated changes have been shown to indicate that microbes can accelerate the aging process (Láng et al., 2024). A fairly consistent finding in studies of age-related diseases is a reduction in microbiome diversity, especially in NDs (Bradley and Haran, 2024). The gut-brain axis (GBA), a bidirectional communication, connects the gut to the brain through various neurotransmitters and metabolites. Multiple biological systems are involved in the GBA, which is essential to maintain the stability of the whole body (Morais et al., 2021). In addition, gut microbiota, with its unique metabolic pathways, produces metabolites that can contribute to the development of diseases (Paik et al., 2022), particularly in lipid and amino acid metabolism, which contribute to the development of NDs (Yan et al., 2022; Augustin et al., 2023). Recently, research has shown that micro-ecological dysregulation of the gut microbiota is also associated with a variety of diseases, particularly the neurodegenerative diseases (Cryan et al., 2019). A large body of literature suggests that gut microbiota affects the development of NDs in many ways, especially in metabolism, and that related studies can provide new targets for preclinical diagnosis and preclinical intervention in NDs.

Metabolism accompanies microorganisms throughout their lives, providing them with the energy necessary for their activities or creating a favorable environment for their survival, and some of these metabolites can have an effect on the human body. Recently, the impact of gut microbiota and its metabolite alteration on NDs has received increasing attention. The gut microbiota metabolites can mediate inflammatory responses (Voigt et al., 2022), produce cytotoxicity, and have a direct effect on neuronal cells. What’s more, metabolites can trigger intestinal inflammation and allow more harmful substances to invade the nervous system (Chang et al., 2020). Therefore, the study of metabolites has gradually become an intervening link in the relationship between gut microbiota and NDs.

In this article, we delineated the basic relationship between gut microbiota and neurodegenerative diseases, highlighting how the metabolism of gut microbiota made a difference to neurodegenerative diseases, also focusing on therapies for NDs based on gut microbiota and providing a landscape for NDs in the area of metabolism of gut microbiota.

## 2 Gut microbiota–brain communications

### 2.1 Gut–brain axis (GBA)

The GBA, which is a bidirectional pathway combining the brain and the gut microbiota and critical site for maintaining homeostasis in the gastrointestinal tract, is involved in several physiological processes (Zhang H. et al., 2022). Gut microbes act as signal factors within the GBA to activate the immune system, promote cytokine production, contribute to gastrointestinal motility and mucin secretion (Villavicencio-Tejo et al., 2023). It has been hypothesized that GBA potentially functions through the vagal nerves, and interacts with the neurologic, endocrine, and immunologic systems. The gut microbiota exists as a parasitic symbiosis in the host, which on the one hand can influence GBA through direct contact or immune stress, and on the other hand, as microorganisms produce microbial hormones and metabolites that can act directly or indirectly by specific metabolic pathways. When considering how the microbiome interacts with the nervous system, the most inevitable conclusion is through the control of host neurotransmitters and/or associated pathways (Forsythe et al., 2014; Strandwitz, 2018).

### 2.2 Barrier system permeability and gut-derived molecules

Made up of intestinal mucosal barrier and blood-brain barrier (BBB), the barrier system permeability is a unique communication pathway. Many environmental factors such as stress, dietary change, and diseases can lead to dysfunction of intestinal mucosal barrier. The BBB is a physiological barrier that divides the central nervous system from the peripheral blood. It is formed of pia mater, choroid plexus, cerebral blood vessels, and astrocytes (Ballabh et al., 2004; Daneman and Prat, 2015; Avila and Perry, 2021). It is indicated that the gut microbiota and associated neurotransmitters and metabolites play an important role in BBB function (Braniste et al., 2014). The blood-brain barrier’s permeability can be impacted by some microbial compounds, such as lipopolysaccharides (LPS) and short-chain fatty acids (SCFAs). By interacting with the BBB, the chemicals of SCFAs can inhibit neuroinflammation and neurodegeneration via having an immediate impact on brain neurons or activating the immune and endocrine systems (Braniste et al., 2014; Harrington, 2015; Aho et al., 2021). The mucosal barrier’s dysfunction and increased blood-brain barrier permeability further promote the release of metabolites, byproducts, and cytokines into the bloodstream and stimulate the expression of toll-like receptors (Rezai-Zadeh et al., 2009; König et al., 2016; Brenchley and Douek, 2012).

LPS is an essential component of the Gram-negative bacteria’s outer membrane, including *Bacteroides* and *Prevotella*. It is a bacterial element which is known for its immune-stimulating properties. When present in excessive amounts, it can lead to systemic inflammation and sepsis in germ-free mice (Miller et al., 2005). Research has discovered that gut microorganisms, such as Bacteroides, can produce significant quantities of LPS. This substance then interacts with Cluster of Differentiation 14 (CD14) and Myeloid Differentiation factor-2 (MD-2) proteins, subsequently activating Toll-like Receptor 4 (TLR4) and resulting in inflammation in mice (Cani et al., 2007). In C57 mice, LPS increases BBB permeability mainly by increasing inflammatory factors such as interleukin-6 (IL-6) and interleukin-9 (IL-9) decreasing the expression of intercellular tight junction proteins such as claudin-5, occludin and zonula occluden-1 (ZO-1) (Feng et al., 2018). The increase in these cytokines can be blocked by indomethacin and Irbesartan. In addition to this, LPS increases BBB permeability by activating monocyte-macrophages (Banks et al., 2015). It has been shown that LPS activation activates the immune response mainly through the NF-κB/MLCK/MLC pathway (Yang et al., 2021). Other studies have shown that the pro-inflammatory transcription factor NFκB can be activated by LPS which is secreted by Bacteroidetes and responsible for the development of AD in microglial cells. The function of NFκB is to stimulate the transcription of pro-inflammatory micro RNAs (miRNAs), thereby activating neuroinflammatory mediators and preventing phagocytosis (Zhao and Lukiw, 2018).

SCFAs, generated in the gut, are saturated fatty acids and are affected by the quantity of fiber consumed. The mechanisms by which SCFAs might influence brain function could possibly be through the immunological regulatory pathway, the endocrine pathway, and the neural factor pathway. By modulating the immune system, SCFAs influence the immunity and barrier function of the intestinal mucosa, thereby enhancing the barrier’s integrity and sustaining the production of mucus. SCFAs are responsible for mediating immunological regulation in the system. They achieve that by controlling the production of cytokines, which in turn affect the proliferation and differentiation of immune cells (Corrêa-Oliveira et al., 2016). This association induces an anti-inflammatory response and decreases the production of pro-inflammatory cytokines (IL-1, IL-6, and tumor necrosis factor-α). Monocarboxylate transporters allow SCFAs to cross the BBB, and the upregulation of tight junction protein expression by SCFAs has been shown to maintain the BBB’s integrity (Vijay and Morris, 2014). In addition, SCFAs have the potential to alter the levels of neurotransmitters as well as neurotrophic factors. According to the research, it’s possible that the bacteria in gut are responsible for either the production of neurotransmitter precursors, or catalyzing of the synthesis and release of a variety of neurotransmitters through the metabolism of food, or both (Chen et al., 2021).

### 2.3 Nervous system modifications

The CNS, autonomic nervous system (ANS), enteric nervous system (ENS), and the hypothalamic–pituitary–adrenal (HPA) axis form the bidirectional communication network. The brain can adjust intestinal activity through the parasympathetic and sympathetic nervous systems, and control digestive secretions and intestinal movement through hormones and neurotransmitters. On the other hand, the gut microbiota also generates molecules that influence the activity of the host immune system and the function of the CNS. The ENS is a network made up of sensory neurons, motor neurons and interneurons of the ANS, which is a quasi-autonomous part of the nervous system. It regulates digestion, intestinal peristalsis and permeability, bile secretion, glucose levels, mucosal mechanical deformation, epithelial fluid level, luminal osmotic pressure, mucus production, and mucosal immunological response (Spencer and Hu, 2020). There are mounting proofs that the gut microbiota may interact with the CNS through metabolites and neurotransmitters with neuroregulatory qualities such as tryptophan, serotonin (5-HT), γ-aminobutyrate (GABA), glutamine, histamine, SCFAs, catecholamines, and others, like the ENS (Ojeda et al., 2021). Short-chain fatty acids are the major metabolites produced by microbial fermentation of dietary fiber (Ikeda et al., 2022). The majority of research has been conducted on acetic acid, propionic acid, and butyric acid. Their molar ratio in the colon is approximately 60:20:20 (Cummings, 1981). SCFAs can serve as an energy source for epithelial cells as well as gut microbiota (Chang et al., 2014; Usami et al., 2008). Additionally, it has been demonstrated to stimulate neurosecretion of neuropeptides and hormones (Rooks and Garrett, 2016). In particular, SCFAs can activate the GPR43/AMPK pathway, which in turn mediates intestinal inflammation and cytokine release (Yang et al., 2018). Enterochromaffin cells in the colon synthesize and secrete serotonin, which is therefore required for physiological processes in the intestine (Yano et al., 2015). However, some bacteria, such as *Corynebacterium spp.*, *Streptococcus spp*. and *Esherichia coil*, also synthesize serotonin in the intestine (Yano et al., 2015). Despite the fact that serotonin does not cross the blood-brain barrier, serotonin can still affect the brain by directly interacting with 5-HT3 receptors on vagal afferent fibers and directly activating the vagus nerve (Raybould, 2010). The HPA axis is a component of the limbic system that includes the amygdala, hippocampus, and hypothalamus, and it plays a role in both the formation of memories and the expression of emotional reactions. When driven by prolonged stress or pro-inflammatory cytokines like IL-6, the levels of corticotropin releasing factor and corticotropin produced by the pituitary gland rise. This results in an increase in cortisol, which is toxic to the brain, being released from the adrenal gland (Frankiensztajn et al., 2020).

### 2.4 Regulation of immune system

**FIGURE 1:**
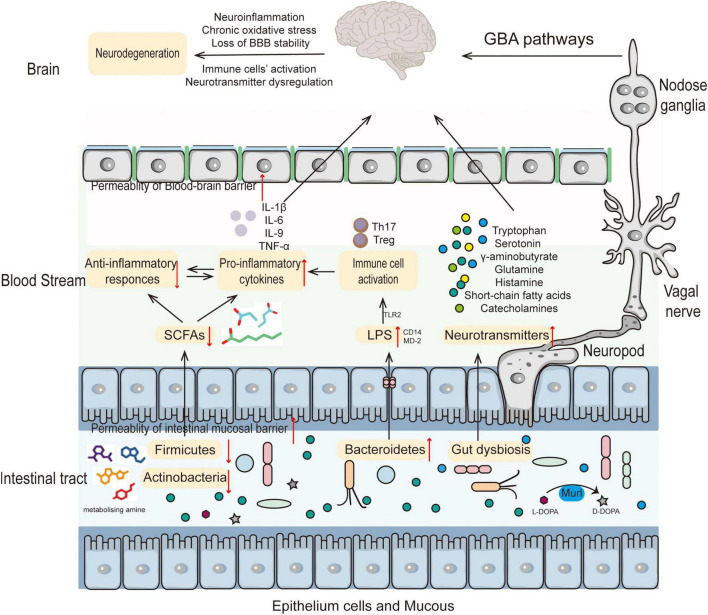
The role of the gut microbiota in the pathogenesis of neurodegenerative diseases. Altered composition of gut microbiota results in decreased SCFAs and increased LPS leading to immune cell activation thereby increasing BBB permeability. Accompanying metabolite changes can cause neurotransmitter dysregulation in the nervous system or directly participate in the pathogenesis of NDs.

The gut microbiota acts out a significant part in the development of the immune system of host as well as the maintenance of intestinal homeostasis (Sommer and Bäckhed, 2013). A disruption in the connection between the immune system and the microbiota might cause an increase in immunological signaling, which may have ramifications for the development of the CNS and NDs (Fung et al., 2017). It has been shown that the gut microbiome regulates Th17 cells and Treg cells, implying that microbiome composition has a remarkable effect on the immune response against pathogenic microorganisms and inflammatory responses (Park et al., 2015). Intricate interactions between gut microbiota and intestinal mucosa boost the host’s cellular immunological response by activating immune cells and releasing cytokines (Ashraf and Shah, 2014). It has been shown that the species of bacteria known as *Lactobacillus salivary* and *Bifidobacterium breve* are regarded to be significant contributors to the maintenance of a healthy immune system (Drago et al., 2015). Other probiotic gut bacteria species, such as *Lactobacillus plantarum*, *Bifidobacterium infantis*, and *Lactobacillus rhamnosus*, should be investigated for their potential anti-allergic and anti-autoimmune benefits (Elian et al., 2015; Mileti et al., 2009; Scaldaferri et al., 2013) (Figure 1).

## 3 The relationship between gut microbiota and NDs

Given that aging is a significant risk factor for NDs, it becomes crucial to initially comprehend how gut microbiota alterations occur in populations as they age. Aging is an unavoidable physiological transition that is accompanied by progressive dysfunction in most tissues and organs including a progressive deterioration in regenerative capacity, cell proliferation, telomere maintenance and genomic stability (López-Otín et al., 2023). There is generally a degree of stability in the adult microbiome, but significant changes in the gut microbiota occur with age, which may be related to physiological changes in the gastrointestinal tract (Rinninella et al., 2019; Zoetendal et al., 1998). A recent study using 16sRNA sequencing revealed that the relative abundance of *Bacteroidota* and *Lactobacillus spp.* decreased in the gut of old mice, whereas the relative abundance of *Alistipes* increased (Donati Zeppa et al., 2022). It has been shown that aging African turquoise killifish, a new model organism, have significantly lower gut bacterial abundance. Significant differences were also found in the gut bacterial composition of young and old fish. The intestines of young fish were enriched with *Bacteroidetes*, *Firmicutes* and *Actinobacteria*, whereas the intestines of old fish were represented by *Proteobacteria* (Smith et al., 2017). Differences in gut microflora are not only found in old versus young animals, but also in people of different ages. One study found that the proportion of *Bifidobacteria* in the gut microbiota of older people over the age of 70 decreased, while the proportion of *Clostridium* and *Proteobacteria* increased, compared to younger people (Odamaki et al., 2016). It has been demonstrated that the diversity of *Bacteroides* species increases in the faces of healthy older adults compared to healthy adolescents. However, with increasing age, *Bifidobacterial* species diversity decreased (Hopkins and Macfarlane, 2002).

**FIGURE 2:**
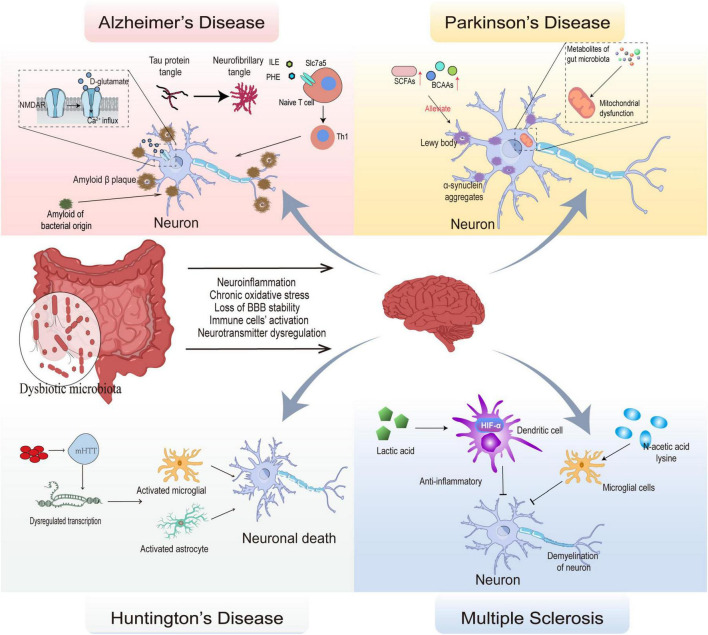
Mechanisms of gut microbial composition and its metabolic alterations in several neurodegenerative diseases. The dysbiotic microbiota affects BBB stability, leads to activation of immune cells, dysregulates neurotransmitters, and promotes oxidative stress, leading to the development of neuroinflammation and neurodegeneration. In Alzheimer’s disease, persistent activation of NMDAR by D-glutamate causes Ca^2+^ influx leading to cytotoxicity; phenylalanine and isoleucine regulate naive T cell differentiation to Th1 via Slc7a5 causing autoimmunity. Eventually, Tau protein tangles accumulate into neurofibrillary tangles and amyloid of bacterial origin act as a prion protein to cause endogenous amyloid production, which ultimately lead to the onset of Alzheimer’s disease. In Parkinson’s disease, α-synuclein aggregation leads to loss of function in dopaminergic neurons, and metabolites of gut microbiota is involved in this process by mediating mitochondrial dysfunction, SCFAs, BCAAs can delay neuronal damage. In Huntington’s disease, aggregation of aberrantly expressed mHTT and activation of microglial and astrocyte leads to neural death. In Multiple sclerosis, autoimmunity mediates neuronal demyelination, and lactic acid inhibits the antigen-presenting role of dendritic cell in autoimmunity by activating HIF-α; N-acetic acid lysine protects nerves by inhibiting microglial immunity.

Loss of neural function over time is a hallmark of neurodegenerative illnesses, which may eventually lead to difficulties with movement and thought. At present, the incidence of neurodegenerative diseases is quickly climbing to epidemic proportions. Despite the fact that having a family history of neurodegenerative disorders is one of the most important risk factors, environmental variables throughout life also have a considerable effect on the start, progression, and final severity of such disorders (Sini et al., 2021). Increasing clinical and experimental data shows that alterations in gut microbes may, to some degree, enhance the risk of neurodegeneration. Behind the altered gut microbiota, the accompanying altered metabolism of the gut microbiota becomes an important factor potentially influencing the development of NDs (Coker et al., 2022). The metabolites of the gut microbiota can not only directly interact with the cells of the intestinal wall to increase the permeability of the intestinal wall, but can also enter the blood stream by absorption and cross the blood-brain barrier to directly and directly act on neurons (Mishra et al., 2023; Figure 2). Therefore, research on the metabolism of gut microbiota can be helpful for the clinical intervention of ND through multi-targets and multi-methods.

### 3.1 Alzheimer’s disease

The most prevalent kind of dementia is Alzheimer’s disease (AD), and it may be identified by increasingly aberrant patterns of both cognitive and behavioral functioning. The key pathogenic characteristics of AD are synaptic damage, abnormal buildup of extracellular beta-amyloid (Aβ), and abnormal formation of neurofibrillary tangles of tau protein. Death of neurons and damage to brain tissues accompany these changes. AD is a brain illness that progresses slowly for many years before symptoms appear (Alzheimers and Dementia, 2022). A great deal of research on Alzheimer’s disease therapy or intervention tactics has shown encouraging outcomes in animal models and clinical trials (Nakamura et al., 2023; Dubois et al., 2023; Wunderlich et al., 2023; Guha et al., 2022). Sadly, medications that target pathogenic processes are ineffective for the clinical therapy of Alzheimer’s disease (Kodamullil et al., 2017). In recent years, a number of preclinical and clinical studies have continuously revealed that the pathogenesis of AD may be directly related to changes in gut microbiome composition (Hoffman et al., 2019; Hill and Lukiw, 2015; Cattaneo et al., 2017). The gut microbiome may play an important role in AD pathology.

#### 3.1.1 The studies of microbiota alteration in AD

Data from animal studies suggested that the gut microbiome conduced to the cognitive impairment and the progression of AD pathology in terms of the aggregation of amyloid and tau, dysregulation of immune system, and neuroinflammation (Kowalski and Mulak, 2019; Wang, 2023). One study found that the abundance of *phylum Proteobacteria* increased and *Firmicutes phylum* decreased in AD mice. This article emphasized an increase in *Proteobacteria* in AD compared to WT mice and a remarkable increase in *Escherichia coli taxa* at the genus level. Similarly, significant changes in the group of *Bacteroidetes* in AD mice were elaborated, including an increase in the *genus Pasteurella* (D’Argenio et al., 2022). These are gram-negative bacteria whose outer membranes are mostly composed of lipopolysaccharides (LPS). It has been well established that LPS is a potent stimulator of the innate immune system in vertebrates (Chen et al., 2024). Moreover, pathogenic alterations in Aβ are linked to the gut microbiota. In a recent study, 3-month-old APPSWE/PS1E9 mice (a model for Aβ amyloidosis) that got gut microbiota from 16-month-old APPSWE/PS1E9 mice had more Aβ plaques, but there weren’t any of these changes in wild-type mice. According to these findings, gut microbial dysbiosis, even while it does not generate Aβ plaques, may promote AD in those who have a genetic predisposition for the condition (Wang et al., 2021). The intestinal microbiota of patients with mild cognitive impairment (MCI), a pre-dementia state, has undergone AD-related changes, indicating that the intestinal microbiota has changed before the onset of AD. The gut microbiome’s α-diversity was significantly reduced in AD patients. Clinical research has shown that elderly individuals with cognitive impairment and brain amyloidosis had less anti-inflammatory *Eubacterium rectale* and *Bacteroides fragilis* and more pro-inflammatory *Escherichia/Shigella spp* (Cattaneo et al., 2017; Mancuso and Santangelo, 2018). Butyrate-producing species like *Faecalibacterium* were dramatically reduced in AD patients, which was positively connected with AD symptoms. What’s more, lactate-producing species such as *Bifidobacterium rose* were adversely linked with clinical symptoms. This dysbiosis of the gut microbiota in AD patients disrupts the pathways involved in folate production and fatty acid metabolism, leading to immunomodulatory dysfunction (Ling et al., 2021). Intriguingly, a scientific trial showed that AD patients’ cognitive, sensory, and emotional capacities were significantly enhanced after ingesting fermented milk containing *Bifidobacterium* and *Lactobacillus spp* (Tillisch et al., 2013).

A growing body of research confirms that alterations in the composition of gut microbiota directly affect cognitive function, which has important consequences for the pathogenesis and progression of AD (Cattaneo et al., 2017; Liu et al., 2019; Mitra et al., 2023). In patients with AD, the accumulation of pro-inflammatory bacteria in the gastrointestinal tract leads to increased intestinal permeability, disruption of the integrity of the blood-brain barrier, and ultimately neuroinflammation (Varesi et al., 2022). Disruption of the blood-brain barrier due to alterations in the gut microbiota allows Aβ peptides, pro-inflammatory factors, and immune cells to enter the brain from the periphery, resulting in changes in the brain’s internal environment that ultimately lead to disease (Kincaid et al., 2021). In addition, it has been found that the application of anti-inflammatory *Bifidobacterium longum* can alter the composition of the gut microbiota and inhibit the expression of amyloid-β, β/γ-secretase, cystatinase-3 and the accumulation of amyloid β in the hippocampus of 5 × FAD-Tg mice thereby alleviating cognitive impairment in AD mice (Lee et al., 2019).

#### 3.1.2 Metabolism of gut microbiota in AD

It has been shown that gut microbiota can increase the ratio of phenylalanine/isoleucine to promote the expression of the phenylalanine and isoleucine transporter Solute Carrier Family 7 Member 5 (Slc7a5), and regulate the differentiation of T cells to Th1 cells in the brain to promote the development of AD (Wang et al., 2019). In the gut microbiota, *Bacteroides spp*. and *Fusobacterium spp*. are capable of producing polyamines, which are mainly spermine, spermidine, and putrescine (Sánchez-Jiménez et al., 2019) and are ligands for various receptors such as N-Methyl-D-Aspartate Receptor (NMDAR) and calcium-sensing receptor (CaR) (Zhang et al., 1994; Hofer and Brown, 2003). Activation of these receptors allows large amounts of Ca^2+^ to be inwardly flowing. Although studies have shown that Ca^2+^ overload occurs after Aβ deposition, however, it has been demonstrated that excess intracellular Ca^2+^ can be taken up by mitochondria to maintain homeostasis, and that when overloaded in mitochondria Ca^2+^ can cause mitochondria to become dysfunctional and play an important role in the process of iron death (Calvo-Rodriguez et al., 2020; Sun et al., 2023). In addition, *Desulfovibrio alaskensis* and *Desulfovibrio desulfuricans* convert choline in the gut to TMA, which selectively activates trace amine-associated receptors (TAARs) (Craciun and Balskus, 2012). More interestingly, trace amine-associated receptors (TAARs) is abundantly ectopically expressed in the brain and are associated with many neurological disorders such as AD, depression, and schizophrenia, and the ability of gut microbiota to metabolize amino acids in the gut to ligands of TAARs such as trimethylamine and cadaverine makes TAARs a potential target for gut microbiota to promote the development of AD (Shang et al., 2023; Guo et al., 2023).

In addition to relying on independent metabolism to produce neurotransmitter-like substances, gut microbiota also produces a number of small-molecule ligands that affect the metabolism of neuronal cells and produce cytotoxicity. It has been found that gut microbiota contains the enzyme Murl, which converts L-glutamate to D-glutamate. D-amino acids are present in trace amounts in mammals. In contrast to L-amino acids, D-amino acids are typically present in lower amounts and are synthesized primarily through food intake and by gut microbiota (Lee et al., 2022). D-glutamate can over-activate NMDAR to produce persistent Ca^2+^ influx in neuronal cells, which in turn produces cytotoxicity (Bertoldi et al., 2003; Choi et al., 1988). More interestingly, it has been found that amyloid of bacterial origin can increase the immune response of the nervous system to endogenous amyloid, more importantly, these bacterial amyloid proteins share tertiary structural similarities with neural amyloid (Friedland and Chapman, 2017), and through molecular mimicry, bacterial amyloid proteins can induce amyloid misfolding like prion proteins, leading to their aggregation and precipitation (Friedland and Chapman, 2017; Friedland, 2015). Currently, there are few studies on gut microbiota-related lipid metabolism, but Xin Cheng et al. found differences in lipid metabolites in feces, serum, and brain between APP1/SP1 and normal mice, implying that enzymes related to lipid metabolism in gut microbiota may play a potential role (Cheng et al., 2022). The variety of lipids that bacteria are able to synthesize is enormous, with the major classes including phospholipids, lipopolysaccharides, and sphingolipids, and thus the resolution of intestinal lipid composition may be an important area of research (Brown et al., 2023). In conclusion, gut microbiota metabolism-related research still has a broad prospect, which is involved in the occurrence and development of AD, and gut microbiota metabolism-related research can provide new targets for the early diagnosis of AD as well as pharmacological interventions in AD.

### 3.2 Parkinson’s disease

Parkinson’s disease (PD) is one of the most common neurodegenerative diseases, and it is anticipated that, by 2040, there will be more than 10 million confirmed cases worldwide (Yang et al., 2022). PD is caused by the death of dopaminergic neurons in the substantia nigra compactus, followed by a loss of dopamine in the striatum (Lotharius and Brundin, 2002). Elevated production and misfolding of α-synuclein, as well as its aggregation to create Lewy bodies, are additional causes of neurodegeneration in PD patients (Olanow and Brundin, 2013; Mahul-Mellier et al., 2020). In addition, PD is thought to be caused by misfolded α-synuclein that starts in the gut and then locates in the brain and PD is strongly associated with gastrointestinal complications including bloating, nausea, and abdominal discomfort (Hayes, 2019; Braak et al., 2003a). Animal models of PD have been shown to have abnormal deposition of α-synuclein in the olfactory bulb or ENS due to changes in gut microbiome composition. These abnormal deposits may be conveyed through the trans-synaptic transmission to the dorsal motor nucleus of the vagus nerve and subsequently through retrograde axons to other sections of the CNS (Peelaerts et al., 2015). These findings indicated that intestinal microbes play a crucial role in the pathogenesis of PD.

#### 3.2.1 The studies of microbiota alteration in PD

Concerning the pathogenesis of α-synuclein, a substantial body of data demonstrates a two-way connection between the gastrointestinal tract and the brain. Accumulation of α-synuclein in neurons may be seen in both the brain and the ENS. As α-synuclein inclusions initially occur in the ENS and then go to the brain through glossopharyngeal or vagal neurons, the gastrointestinal tract may accelerate the spread of Parkinson’s disease (Braak et al., 2003b; Shannon et al., 2012). Holmqvist et al. demonstrated that α-synuclein travels from the intestines to the brain through the vagal nerves (Holmqvist et al., 2014). Nevertheless, Arotcarena et al. could not discover any pathological lesions caused by α-synuclein in the vagal nerve. This disproves the hypothesis that the vagal nerve plays a role in the spread of α-synuclein pathology (Arotcarena et al., 2020). In another study, patient-derived α-synuclein aggregates injected into the enteric or striatum of baboon monkeys caused nigra striatum damage and ENS pathology, demonstrating the gut-brain axis’ participation in Parkinson’s disease transmission. Pathological alterations in the dorsal motor nucleus of vagal nerves, locus coeruleus, amygdala, dorsal raphe nucleus, and substantia nigra pars compacta were created in mice by injecting α-synuclein fibrils into their intestines, mimicking the motor and non-motor symptoms of PD (Kim et al., 2019). Additionally, nigral overexpression of α-synuclein resulted in a significant loss of neuronal cells in the ileal submucosal plexus, as well as changes in the microbiota of the gut and the metabolism of bile acid (O’Donovan et al., 2020). Mice that overexpressed α-synuclein and were either treated with antibiotics or maintained in sterile circumstances exhibited enhanced motor capabilities and a decreased number of α-synuclein deposits. Fecal microbiota transplantation (FMT) from Parkinson’s patients to mice that overexpress α-synuclein resulted in a greater decline in motor function than the transplantation of gut bacteria from healthy individuals, which suggests that changes in the gut microbiota are the cause of disease symptoms (Sampson et al., 2016).

It has been shown beyond a reasonable doubt that persons with Parkinson’s disease have significantly distinct gut flora from healthy individuals. The relative abundance of *Clostridium family XI* and the *Bacillus alimentary canal* in PD patients was high, while the numbers of genera *Faecalis* and *Clostridium* were reduced (Weis et al., 2019). Interestingly, the gut microbiota of PD patients who received medication also differed from that of healthy control group. The relative abundances of the *Peptoniphilus* and *Finegoldia* genera were significantly increased in PD patients treated with levodopa, whereas the relative abundances of *Peptophagus*, *anaerobic cocci*, *Euobacteria brevis*, *Sellimonas*, *Bifidobacteria*, and *Enterococcus* were increased in PD patients treated with Entacapone (Weis et al., 2019). It should also be emphasized that the human intestine includes both bacteria and unidentified eukaryotes. Recently, it has been found that eukaryotic abundance is reduced in PD patients. At the same time, when PD patients were given levodopa or Entacapone, the abundance of *Geotrichum rose* in comparison to healthy controls (Weis et al., 2021).

#### 3.2.2 Metabolism of gut microbiota in PD

More and more studies have begun to focus on the potential mechanisms by which microbial changes in the gut microbiota led to the development of PD, and the metabolite changes accompanying gut microbiota alterations may be an important factor in the development of PD. Sampson et al. enabled the oral administration of specific microbial metabolites to sterile mice to be able to trigger neuroinflammatory and locomotor symptoms (Sampson et al., 2016). Branched-chain amino acids (BCAAs) were found to significantly ameliorate locomotor symptoms and reduce the loss of dopaminergic neurons in PD mice by decreasing the secretion of IL-6, IL-1β, and TNF-α from immune cells (Yan et al., 2022). BCAAs attenuate the levels of inflammatory factors (IL-6, IL-1β, and TNF-α) to delay the progression of PD in a fisetinone-induced mouse model. These molecules activate the NF-κ pathway in microglia, which promotes the activation of nucleotide-binding oligomerization domain-like receptor protein 3 (NLPR3) inflammatory vesicles, leading to chronic neuroinflammatory damage in neurons (Fan et al., 2020). However, gut microbiota alterations were associated with decreased plasma concentrations of leucine, isoleucine, valine, and tyrosine in PD patients and worsened with the severity of PD symptoms (Zhang Y. et al., 2022). In lipid metabolism, Erny et al. demonstrated that gut microbiota-synthesized SCFAs, especially acetic acid, can regulate microglia maturation and function, and repair microglia damage by incorporating microglia core metabolism (Erny et al., 2015). Energy metabolism related studies are fewer, but through macro-genome sequencing and metabolomics techniques, it was verified that gut microbiota produces metabolites associated with mitochondrial dysfunction and that it is implicated in the pathogenesis of PD (Yan et al., 2021). The potential influence of gut microbiota in other metabolisms has also been found, with studies finding that the gut microbiota of PD patients is capable of producing indole and homocysteine (Rosario et al., 2021) and that *Enterococcus* intestinalis reduces the efficacy of levodopa by degrading it via tyrosine decarboxylase (Maini Rekdal et al., 2019).

### 3.3 Huntington’s disease

Brain shrinkage, most noticeably in the cerebral cortex and striatum, is a hallmark of Huntington’s disease (HD), a genetic neurodegenerative condition. These people have progressively deteriorated muscular, cognitive, and psychological symptoms over the course of 15–20 years until they eventually succumb to their illness and die. HD patients are diagnosed after the onset of significant motor symptoms, which usually occur between the ages of 35 and 55, although other symptoms can appear 10–15 years earlier (Bates et al., 2015). The increase of CAG repeats in the Huntington protein (Htt) gene is the cause of the condition. This gene is ubiquitously expressed, meaning it may be found not just in the brain but also in many other tissues and cells throughout the body, such as skeletal muscles and gut intestinal epithelial cells (The Huntington’s Disease Collaborative Research Group, 1993; Sathasivam et al., 1999; Moffitt et al., 2009). Transcriptional regulation and normal cell function are profoundly disrupted by the expression of HD mutations, thereby affecting overall physiology (Chang et al., 2006; Kim et al., 2010; Luthi-Carter and Cha, 2003). As a result, people who have this condition also suffer from a variety of peripheral deficits, such as a wasting away of their skeletal muscles, a major loss of weight, and an impaired immunological response (Zielonka et al., 2014; Aziz et al., 2008; Björkqvist et al., 2008). Prior research has shown that individuals with HD and transgenic animals both had different levels of circulating gut metabolites, which suggests that the gut microbiota may shift even before the beginning of the illness (Verwaest et al., 2011).

#### 3.3.1 The studies of microbiota alteration in HD

A recent study that looked at the differences in the gut microbiota of HD mice and wild-type mice found that male HD mice had a greater number of *Bacteroidetes* and *Lactobacillus* but a lower abundance of *clostridium* in their guts. On the other hand, female HD mice showed a decreased number of *Clostridiales* and an increased number of *Coriobacteriales*, *Erysipelotrichales*, *Bacteroidales*, and *Burkholderiale*. Additionally, male HD mice exhibited a greater variety of microorganisms than female HD mice as well as wild-type mice (Kong et al., 2020). Commensal fungus, much like intestinal bacteria, contribute significantly to the regulation of the host immune system and metabolic functions (Chiaro et al., 2017; Paterson et al., 2017; Underhill and Iliev, 2014; Jiang et al., 2017). Recent research has shown that the fungus community in the gut may cause significant changes in the organization of the gut bacteriome and help mold the gut microbiome throughout the formative years of an organism’s existence (van Tilburg Bernardes et al., 2020). These fungal groups contributed to the development of the host immune system by acting synergistically with the gut bacterial community to trigger a powerful local and systemic immune response (van Tilburg Bernardes et al., 2020; Leonardi et al., 2018). Moreover, several fungal species are commonly employed as probiotics due to their ability to release enzymes that neutralize toxins produced by inflammatory intestinal residents and prevent the growth of additional possible pathobionts (Markey et al., 2018; Buts et al., 2006; Kumamoto et al., 2020). The abundance of numerous important fungal species was also found to have changed. In addition, in the HD mice by 12 weeks of age, *Malassezia restricta* and *Yarrowia lipolytica* were underrepresented in the gut mycobiome (Kong et al., 2022). Between the patients with HD and the healthy control group, the study found several significant changes in the fecal microbiota. To be more specific, the relative abundances of the following in the patients with HD were considerably greater than those in the group with healthy controls: *Actinobacteria*; *Deltaproteobacteria Actinobacteria*; *Desulfovibrionales*; *Oxalobacteraceae*; *Lactobacillaceae*; *Desulfovibrionaceae*, and so on. In contrast, the healthy control people had significantly higher levels of *Clostridium XVIII* at genus than that in the HD group (Du et al., 2021).

#### 3.3.2 Metabolism of gut microbiota in HD

Although HD is a degenerative disease caused by polyglutamine amplification due to genetics (The Huntington’s Disease Collaborative Research Group, 1993), metabolites of the gut microbiota can have an impact on the timing of the onset of motor symptoms in HD. It has been shown that sodium butyrate improves the neurodegenerative phenotype of HD, which may delay the onset of motor symptoms (Ferrante et al., 2003). HD patients express large amounts of mutant huntingin that bind to and inhibit histone acetyltransferases, resulting in blocked transcription of genes, and sodium butyrate, an inhibitor of histone deacetylases, restores gene transcriptional activity to slow the progression of HD (Steffan et al., 2000; McCampbell et al., 2001). Sodium butyrate enhances specificity protein-1 acetylation and inhibits 2,3-nitropropionic acid-induced excitotoxicity (Ryu et al., 2003) and improves oxidative metabolism by increasing histone acetylation at the transcriptional level to promote bead protein expression (Ferrante et al., 2003; Sealy and Chalkley, 1978). In amino acid metabolism, kynurenic acid (KYNA), a metabolite of tryptophan, is able to delay neurodegenerative changes in HD (Campesan et al., 2011). KYNA acts as a competitive inhibitor of the glycine agonist site in the NMDAR and a non-competitive inhibitor of the α7-nicotinic acetylcholine receptor (Alkondon et al., 2011), and inhibits the release of glutamate within physiological ranges; thus, KYNA may be neuroprotective by antagonizing these receptors and reducing glutamate-dependent neurotoxicity (Goda et al., 1999; Zwilling et al., 2011).

### 3.4 Multiple sclerosis

Multiple sclerosis (MS), a chronic neurological disease of CNS, is immune-mediated and characterized by demyelination and damaged axons that affects around 2.3 million individuals worldwide. Females are more likely to be affected (Doshi and Chataway, 2017; Ochoa-Repáraz et al., 2018; Harbo et al., 2013). MS presents different phenotypes, with primary progressive multiple sclerosis (PPMS) accounting for around 15% of cases and relapsing-remitting multiple sclerosis (RRMS) accounting for 85% (Vidal-Jordana and Montalban, 2017). The pathologic feature of MS is the formation of inflammatory localized demyelinated plaques in the CNS, which may occur in the gray or white matter of the spinal cord and the brain. A neuroinflammatory response is triggered by these plaques, which in turn leads to the demyelination of neurons and other specialized cells and ultimately neurodegeneration. Various cells of the immune system infiltrate into CNS neurons, resulting in demyelination as a result of the abnormal permeability of BBB. Myelin antigen-specific T cells, including CD8^+^ and CD4^+^ T cells, are able to pass through this barrier, which contributes to a chain of events that ultimately results in the development of demyelinating lesions (Frohman et al., 2006). Recent research has shown that the commensal microbial communities in the gut are also to blame for a number of immune-mediated diseases like MS and can be thought of as a new environmental risk factor. In other words, immunomodulation, alterations in BBB integrity and function, promotion of the autoimmune demyelinating process, and direct interaction with a wide variety of CNS-resident cell types are all functions mediated by the gut microbiota (Schepici et al., 2019).

#### 3.4.1 The studies of microbiota alteration in MS

When transgenic mice expressing genes encoding a rearranged T cell receptor specific for myelin basic protein were housed in a non-sterile facility but not in a specific-pathogen-free environment, they developed spontaneous experimental autoimmune encephalomyelitis (EAE), an animal model of autoimmune and inflammatory diseases including MS. This provided early evidence that bacteria may play a role in CNS autoimmunity (Goverman et al., 1993). The initial indication that commensal bacteria have a role in neurological autoimmune illness originated from antibiotic-induced reductions in the natural gut microbiota. Antibiotic treatment reduced the level of TH17 cells in the mesentery, leading to a reduction in the severity of EAE (Yokote et al., 2008). This impact required a fraction of invariant natural killer cells, indicating that innate immune processes regulate CNS autoimmunity through microbes. Another groundbreaking work found that oral antibiotic therapy protected against actively generated EAE, suggesting that the medication either downregulated pro-inflammatory or elevated anti-inflammatory pathways (Ochoa-Repáraz et al., 2009). Later research revealed commensal *Bacteroides fragilis* as protective bacteria that inhibits EAE by producing capsular polysaccharide A, which activates T reg cells through the Toll-like receptor 2 signaling pathway (Round et al., 2011; Wang and Kasper, 2014; Ochoa-Repáraz et al., 2010). Recent research has shown that the gut microbiota has a substantial effect on MS and may be altered by environmental variables (Schepici et al., 2019). When comparing MS patients to healthy controls, 16S rRNA sequencing of the gut microbiome indicated that MS patients exhibited a greater abundance of the phylum *Firmicutes* and a lower abundance of the phylum *Bacteroidetes* (Cosorich et al., 2017). Untreated MS patients also have a greater abundance of *Euryachaetota* and *Akkermansia* than healthy control patients (Jangi et al., 2016). In particular, other studies have suggested that reduced *Prevotella* abundance in RRMS patients increases disease activity (Cappello et al., 2016). A decrease in the number of *Clostridium* bacteria in RRMS patients, leading to a decrease in SCFAs secretion levels, was also observed in one study (Miyake et al., 2015). FMT experiments have provided more evidence that there is a connection between changes in the gut microbiota and MS by examining the magnitude of such alterations in individuals who have the disease. Transplanting the MS microbiome in a mouse model resulted in an increased incidence of experimental autoimmune encephalomyelitis, and resulted in more severe MS symptoms (Berer et al., 2017; Cekanaviciute et al., 2017).

#### 3.4.2 Metabolism of gut microbiota in MS

Studies have shown that metabolites of *Lactobacillus* play an important role in the development of MS. Lactic acid produced by *Lactobacillus* is able to activate hypoxia-inducible factor 1α (HIF-α) thereby inhibiting dendritic cells-mediated autoimmune responses (Sanmarco et al., 2023). In addition, its production of N-acetic acid lysine has an inflammatory inhibitory effect on microglial cells, thereby protecting the nerves from immune attacks. *R. gnavus* is able to decarboxylate phenylalanine into phenethylamine (Zhai et al., 2023; Williams et al., 2014). Phenylethylamine was found to hyperactivate dopamine receptor D2 (DRD2) by metabolome precision analysis technique thereby decreasing lysozyme sensitive *Lactobacillus* (Peng et al., 2023). Bacteria, such as *Bacteroides spp*. and *Peptostreptococcus spp.* (Agus et al., 2018), in the gut degrade tryptophan and phenylalanine to phenol and indole derivatives thereby inducing the onset of neurotoxicity in MS (Ntranos et al., 2022). Metabolites (p-cresol sulphate, indoxyl sulphate and N-phenylacetylglutamine) originating from gut microbiota are distributed across the blood-brain barrier into the cerebrospinal fluid. These metabolites differ from other indoles in that they do not rely on neuronal oxidation to inhibit electrophysiological activity and function of neurons, but directly induce neural axonal damage (Ntranos et al., 2022; Pappolla et al., 2021). Other metabolites such as p-cresol sulfate, indole phenol sulfate have also been implicated in the development of MS (Ntranos et al., 2022). The development of MS has been associated with the development of a variety of metabolic pathogens such as p-cresol, indole phenol sulfate, and p-cresol sulfate (Peng et al., 2023).

## 4 Therapies for NDs based on gut microbiota

### 4.1 Fecal microbiota transplantation (FMT)

Fecal microbiota transplantation (FMT) is regarded as an investigational and potential therapeutic treatment for NDs. This is a relatively new treatment. It involves the transplantation of microbes and metabolites from the gut of a healthy donor to the gut of a recipient using a fecal sample as a medium (Zhernakova et al., 2016). The healthy microbiota replaces itself via reproduction and creates beneficial compounds through FMT, which is carried out using endoscopies, enemas, and freeze-dried materials. FMT is thought to be safe, even for people who are at high risk (Adenzato et al., 2019; Wimo et al., 2017). In fact, cognitive impairment, amyloid accumulation, and circulating levels of pro-inflammatory markers were all reduced in AD animal models when healthy fecal microbiota was transplanted from wild-type mice (Kim et al., 2020). Similar results were obtained in another experiment. In addition, the experiment also found that after bacterial colonization, the synaptic plasticity of AD model mice was improved, and the bacterial population that produced SCFAs in the intestine increased (Sun et al., 2019). The therapeutic effect of FMT in animal models of NDs has been confirmed by a large number of experiments, however, only two human case studies have shown promising results (Hazan, 2020; Park et al., 2021). According to Hazan et al., an 82-year-old man who got FMT from his wife (an 85-year-old woman) improved his AD symptoms such as cognitive function, memory, and mood (Hazan, 2020). After treated with FMT by a healthy 27-year-old man, a 90-year-old lady with AD and severe *Clostridium difficile* infection demonstrated improvements in cognitive function, microbiome diversity, and SCFA synthesis in the second case study (Park et al., 2021). Numerous research on FMT on different neurological illnesses have been conducted, and more trials are still underway. However, the safety problems faced cannot be ignored when FMT is applied to clinical practice (Hediyal et al., 2024). In the United States, six cases of serious infections following FMT treatment have been reported by the Food and Drug Administration (FDA, 2020). Therefore, it is essential to set up relevant clinical implementation protocols that enable clinicians to operate with the highest degree of quality and safety assurance, and these behaviors can minimize the risks associated with the process (Table 1).

**TABLE 1:** Neuroprotective effects of FMT in neurodegenerative diseases.

Interventions	Type	Subjects	Donors	Results	References
FMT	Preclinical researches	AD-like pathology with amyloid and neurofibrillary tangles (AD*^LPAPT^*) transgenic mouse model of AD	Healthy wild-type (WT) mice	Improved cognitive impairment, reduced amyloid accumulation and circulating levels of pro-inflammatory markers	Kim et al., 2020
		APPswe/PS1dE9 transgenic (Tg) mouse model	Healthy wild-type (WT) mice	Improved cognitive impairment, reduced amyloid accumulation and tau expression, enhanced synaptic plasticity, and increased SCFAs-producing bacteria in the gut	Sun et al., 2019
	Human case studies	An 82-year-old man presented as a gradual 5-year decline in memory and cognition	The patient’s 85-year-old wife	A marked improvement in mood, was more interactive, and showed more expressive affect	Hazan, 2020
		A 90-year-old woman with Alzheimer’s dementia	A 27-year-old man with no gastrointestinal or other health problems, not using drugs and antibiotics	A marked improvement in mood (GDS 17) and daily living activities and showed more expressive affection	Park et al., 2021

### 4.2 Probiotic

A variety of therapeutic interventions, including the use of probiotics, have been used to treat intestinal microbiome disorders with the aim of restoring intestinal microbiome balance and improving clinical outcomes in neurological diseases (Kerry et al., 2018). *Bifidobacterium* and other lactic acid-producing bacteria, such as *Lactobacillus*, make up the bulk of a probiotic’s composition. In addition, the use of probiotics in the treatment of human neurodegenerative diseases, such as AD, has shown encouraging outcomes. First, Lactobacillus plantarum was reported to improve cognitive performance in AD mouse models and increase levels of acetylcholinesterase in the brain (Nimgampalle and Kuna, 2017). In a different research, rats that had been injected with Aβ over a period of eight weeks were given a probiotic combination that included the bacteria strains *Lactobacillus acidophilus*, *Lactobacillus fermentum*, *Bifidobacterium lactis*, and *Bifidobacterium longum*. The findings showed that altering the makeup of the gut microbiome enhanced spatial memory, decreased learning deficits, and lowered oxidative stress (Athari Nik Azm et al., 2018). Additionally, in a hereditary mouse model of PD, long-term probiotics using six bacterial strains reduced motor deficits and protected dopaminergic neurons (Hsieh et al., 2020). Akbari et al. showed that after taking probiotic milk containing *Lactobacillus acidophilus*, *Lactobacillus casei*, *Bifidobacterium bifidum*, and *Lactobacillus fermentum* for 12 weeks, AD patients had better cognitive performance and insulin metabolism than the control group, but oxidative stress, inflammation, blood glucose, fasting, and indicators of lipid distribution did not change (Akbari et al., 2016). There is also evidence that probiotics are beneficial for PD patients (Castelli et al., 2021). In a randomized controlled trial, patients with PD who took probiotics had improved symptoms compared to controls, as evidenced by reduced scores on the Movement Disorders Society-Unified Parkinson’s Disease Rating Scale (Tamtaji et al., 2019; Table 2).

**TABLE 2:** Neuroprotective effects of probiotic in neurodegenerative diseases.

Interventions	Type	Subjects	Drugs Taken	Results	References
Probiotic	Preclinical researches	D-Galactose-induced Alzheimer’s disease in albino rats.	Lactobacillus plantarum MTCC1325	Ameliorated cognition deficits and restored ACh and the histopathological features	Nimgampalle and Kuna, 2017
		Alzheimer rats, which received an intrahippocampal injection of amyloid (Aβ1–42)	A probiotic mix containing Lactobacillus acidophilus, Lactobacillus fermentum, Bifidobacterium lactis, and Bifidobacterium longum	Improved spatial memory and learning disability, reduced oxidative stress	Athari Nik Azm et al., 2018
		Transgenic MitoPark PD mice	Probiotics consisted of six bacterial strains (Bifidobacterium bifidum, Bifidobacterium longum, Lactobacillus rhamnosis, Lactobacillus rhamnosus GG, Lactobacillus plantarum LP28, and Lactococcus lactis subsp. Lactis)	Had neuroprotective effects and alleviated the progressive deterioration of motor functions	Hsieh et al., 2020
	Human case studies	People with AD (60–95 years old)	Probiotic milk containing Lactobacillus acidophilus, Lactobacillus casei, Bifidobacterium bifidum, and Lactobacillus fermentum	Had a positive effect on the cognitive function and insulin metabolism	Akbari et al., 2016
		Sixty individuals with PD, aged 50-90 years	Probiotic containing Lactobacillus acidophilus, Bifidobacte rium bifidum, Lactobacillus reuteri, and Lactobacillus fermentum	Reduced scores on the Movement Disorders Society-Unified Parkinson’s Disease Rating Scale	Castelli et al., 2021

### 4.3 Drugs target on metabolism

Probiotic interventions were found to reduce neuroinflammatory responses and Aβ deposition, but did not modulate levels of inflammatory factors such as IL-6, IL-10 and glutathione in AD patients (Akbari et al., 2016; Agahi et al., 2018). GV-971, the first new AD therapeutic drug targeting the brain-gut axis, has been newly marketed in China, and it works by inhibiting the accumulation of phenylalanine and isoleucine thereby achieving solid cognitive improvement and reversing cognitive impairment. However, no large-scale clinical studies have been conducted with drugs other than GV-971, but the introduction of GV-971 expands and clarifies the research idea of regulating gut microbiota to prevent and treat AD. Therefore, more research should focus on the pathogenesis of gut microbiota and AD, providing new ideas for the development of more drugs targeting AD. Engineered Clostridium butyricum not only consistently expresses glucagon-like peptide-1(GLP-1) for PD treatment, but also mediates mitochondrial autophagy for neuroprotective effects (Wang et al., 2023). Inspired by this, some beneficial metabolites or key enzymes of metabolism can also be introduced by engineering bacteria for therapeutic effects (Table 3).

**TABLE 3:** Neurological effects of gut microbiota metabolites.

Disease	Type	Metabolite	Effects	References
AD	Preclinical researches	Phenylalanine and Isoleucine	Acting on Slc7a5 mediates neuroinflammation	Wang et al., 2019
	Preclinical researches	D-glutamate	D-glutamate overactivation of NMDAR mediates cytotoxicity	Bertoldi et al., 2003; Choi et al., 1988
	Preclinical researches	Heterogenic amyloid	Involvement in amyloid deposition	Friedland and Chapman, 2017
PD	Preclinical researches	Branched-chain amino acids	Improvement of motor symptoms and reduction of dopaminergic neuronal damage	Yan et al., 2022
	Preclinical researches	SCFAs	Regulation of microglia maturation and function and repair of microglia damage	Weis et al., 2021
HD	Preclinical researches	Kynurenic acid	Delaying neurodegenerative changes in Huntington’s Disease	Campesan et al., 2011
MS	Preclinical researches	Lactic acid	Inhibiting autoimmune by activating HIF-α of dendritic cell	Sanmarco et al., 2023
	Preclinical researches	N-acetic acid lysine	Inhibiting immune reaction of microglia cell	Sanmarco et al., 2023
	Preclinical researches	Indole derivatives	Neurotoxicity	Ntranos et al., 2022

### 4.4 Others

In addition to the most common FMT and probiotic therapies, prebiotics are also a type of gut flora-based therapy used to treat neurodegenerative diseases. Prebiotics have been shown to significantly reduce cognitive and psychological dysfunction in 5xFAD mice, which can be explained in part by altering the gut microbiota and enhancing the formation of SCFAs (Liu et al., 2021). There is also a substance known as synbiotic, a mixture of prebiotics and probiotics, which has been shown to have a regulatory effect on the intestinal microbiome (Lorente-Picón and Laguna, 2021). In addition to pharmacological treatments, changes in diet, appropriate physical exercise and improved sleep quality can also be used to modify the microbial composition of the gut and the abundance of SCFAs and intestinal metabolites, thereby improving the symptoms of neurodegenerative diseases (Wilson et al., 2020; Voigt et al., 2016; Allen et al., 2015; Allen et al., 2018). It has been suggested that a healthy diet is neuroprotective and slows the progression of NDs. Conversely, poor dietary habits can exacerbate neurodegeneration in the elderly (Fernández-Sanz et al., 2019). In 5 × FAD mice, a high-fat diet significantly promoted AD-related pathological changes by increasing oxidative stress and cerebral amyloid angiopathy (Lin et al., 2016). However, a Mediterranean ketogenic diet intervention in patients with MCI found that the ketogenic diet reduced the abundance of *Bifidobacterium* spp. and *Bradyrhizobium* spp. and increased levels of propionic acid and butyric acid (Chen et al., 2020). What’s more, polysaccharides, phytosterols, alkaloids, terpenoids, and carotenoids in marine natural products can bi-directionally regulate the GBA axis and exert neuroprotective effects (Fakhri et al., 2021). It has been confirmed that a high intake of branched-chain amino acids may well improve the motor symptoms of PD (Yan et al., 2022). Therefore, branched-chain fatty acids can be used as a dietary supplement in a way to treat PD.

## 5 Conclusion

The gut microbiota may impact brain diseases in a number of different ways, including the modulation of the immune system, direct neuronal signaling and activation of the humoral route through microbial chemical. The composition of microbiota has changed in neurodegenerative diseases, which can be confirmed in both preclinical and clinical studies. The metabolism of the gut microbiota is altered along with the altered gut microbiota. The production of SCFAs is reduced following alterations in the gut microbiota, and SCFAs are recognized as a protective substance that plays a protective role in the neuroinflammatory response as well as in intestinal permeability. In addition, some fatty acid membrane receptors, such as free fatty acid receptor 4 (FFA4, also known as GPR120), G protein-coupled receptor 41 (GPR41), and G protein-coupled receptor 43 (GPR43), are also involved in this process, allowing drugs targeting lipid receptors to be targeted for the treatment of NDs. Amino acids and their metabolites readily cross the blood-brain barrier and act as ligands for many membrane receptors, so the study of amino acids and their metabolites can be a target for clinical drug intervention. Many enzymes of amine metabolism are contained in gut microbiota, which can metabolize amino acids efficiently, and alterations in gut microbiota led to changes in the composition of amino acids in the gut, thus contributing to the progression of NDs. Dietary supplements of amino acids can play a therapeutic role here. In other metabolic aspects, which are also the most promising, the gut microbiota can process primary bile acids to produce hundreds of secondary bile acids, and the composition of these bile acids can be used as a map to indirectly reflect the composition of the gut microbiota, and at the same time, the study of the mapping of the secondary bile acids may be useful to assist in the early diagnosis of the disease. In conducting this review, our primary goal was to compile the available evidence on alterations in gut microbiota and their metabolic alterations in relation to neurodegenerative diseases, and to explore promising research directions for gut microbiota research. However, a special limitation of this review is its narrow scope, focusing on common neurodegenerative diseases, and not involving some rare diseases. The research on the role of intestinal microbiota in neurodegenerative diseases is still at an early stage, but many studies have pointed out the potentially important role of various microbiota-related therapies in altering the composition of the gut microbiota, and the effects of altered metabolites of the gut microbiota on degenerative diseases are gradually being emphasized, which will provide new ideas for the clinical prevention of and intervention in neurodegenerative diseases.
